# Socio-economic and food system drivers of nutrition and health transitions in The Gambia from 1990 to 2017

**DOI:** 10.1016/j.gfs.2023.100695

**Published:** 2023-06

**Authors:** Zakari Ali, Pauline F.D. Scheelbeek, Sarah Dalzell, Genevieve Hadida, Alcade C. Segnon, Sulayman M'boob, Andrew M. Prentice, Rosemary Green

**Affiliations:** a Nutrition and Planetary Health Theme, MRC Unit the Gambia at the London School of Hygiene and Tropical Medicine, Banjul, Gambia; b Faculty of Epidemiology and Population Health, London School of Hygiene and Tropical Medicine, London, UK; c Centre on Climate Change and Planetary Health, London School of Hygiene and Tropical Medicine, London, UK; d MRC Epidemiology Unit, University of Cambridge, Cambridge, UK; e CGIAR Research Program on Climate Change, Agriculture and Food Security (CCAFS), International Crops Research Institute for the Semi-Arid Tropics (ICRISAT), Bamako, Mali; f Alliance of Bioversity International and International Center for Tropical Agriculture (CIAT), Dakar, Senegal; g Faculty of Agronomic Sciences, University of Abomey-Calavi, Cotonou, Benin; h Kombo Farms, Banjul, Gambia

**Keywords:** Food supply, Micronutrient deficiency, Overnutrition, Under nutrition, Urbanization, Agriculture, The Gambia

## Abstract

In common with many nations undergoing a nutrition transition, micronutrient deficiencies, undernutrition and overnutrition coexist in The Gambia. Addressing these challenges simultaneously would require transformational changes in the country's food system. However, the evidence base that would enable informed decision-making in the Gambian food system has been scant, despite several sources of routinely-collected data being available. This descriptive study brings together data from four open-access global databases on food supply, political, economic, and demographic variables, and nutrition and health between 1990 and 2017 to study potential leverage points for improvement in the food system. It compares trends in food supply and nutritional outcomes in The Gambia against regional and global averages, and identifies potential drivers taken from a food systems framework. The data show that, over the past three decades, total energy supply has increased, and obesity is rising quickly, but iron deficiency persists in a proportion of the population. Overall diet composition is poor, with lower availability of fruit and vegetables and higher supply of sugar and oils compared to regional and global averages. Domestic production is low for most food groups and so a high dependence on imports from other countries bridges the gap in terms of energy supply. Measures of economic development, particularly GDP, were positively related with supply of cereals and animal source foods over time, but no such relationship was observed with fruit and vegetable supply. Food system policy to improve nutrition and health outcomes in The Gambia needs to focus on improving the diversity of food supply – especially fruit and vegetables - and maximizing national domestic production to reduce reliance on food imports. The use of open-source global datasets can be feasible in exploring food system characteristics and trends at the national level and could be applied in other contexts.

## Highlights

• There is utility in combining large datasets on food systems for nutrition and health policy in LMICs.
• Decades of Gambian food system data show adequate gross energy supply but micronutrients are suboptimal.
• Food imports have complemented low domestic production for most food groups in The Gambia.
• GDP closely tracked availability of cereals and animal source foods over time but not fruit and vegetables.

## 1 Introduction

Globally, progress is being made in the fight to address undernutrition but for many countries rates of nutritional deficiency, stunting and related mortality remain high (Development Initiatives, 2020). In 2020, the Africa region had the highest per-capita hunger levels in the world according to the Global Hunger Index (Global Hunger Index, 2020), with more than one in five people classified as undernourished. Transformational changes in the food system are urgently needed to deliver adequate and sustainable foods for all, to continue the fight against hunger and to combat the epidemic of nutrition-related chronic disease that has spread from high income to lower income countries. Global studies of food supply have shown that while many food systems have changed radically in recent decades, those in Africa have tended to show little change (Bentham et al., 2020).

The main concern of diets in Africa is continued pockets of food insecurity together with high rates of micronutrient deficiency (Beal et al., 2017; FAO et al., 2021). West Africa is no exception to these nutritional challenges (Chadare et al., 2022), with the prevalences of undernourishment, stunting in children <5 years old, and anemia in women of reproductive age estimated at 19%, 31% and 52% respectively in 2020 (FAO et al., 2021). A further concern is the nutrition transition, characterized by reductions in undernutrition (largely in children) with corresponding rises in overnutrition among adults (Popkin et al., 2012) – such as a reported 3.0 and 6.6 percentage points increase in obesity in adult men and women respectively in 2016 as compared to 2000 (Development Initiatives, 2020). With these trends, the region is currently at increased risk of facing the “double burden” of malnutrition (Ahinkorah et al., 2021; Prentice, 2018) with countries having to deal with the two extremes of nutritional challenges. In countries such as Ghana and The Gambia, this is already noticeable in national statistics. Approximately 1 in 5 children are stunted in both countries and nearly half the women of reproductive age suffer from anemia. Furthermore, prevalence of overweight is ∼40% for adult women in both countries – coexisting with anemia (Development Initiatives, 2021b; Petry et al., 2019).

Recent government policies in Gambia seek to address the double burden of malnutrition and diet-related non-communicable diseases (NCDs) through complementary and multi-sectoral actions. There is a national nutrition policy (2021–2025) with a broader scope seeking to address all forms of malnutrition and diet-related NCDs (Nattional Nutrition Agency, 2022). The national NCDs strategy and action plan (2022–2027) specifically targets NCD prevention and reduction with plans to increase the availability, affordability, accessibility, and consumption of healthy foods such as fruits and vegetables (Ministry of Health The Gambia, 2022). The agriculture and natural resources policy (2017–2026) complements these through its overall goal of maximizing poverty reduction and enhancement of income, food and nutrition security through optimal utilization of resources without compromising the integrity of the environment (Ministry of Agriculture The Gambia, 2017).

The implementation of policies to prevent and reduce the double burden of malnutrition and diet-related NCD needs urgent attention. To be effective, a food systems approach with a shift in paradigm: rather than feeding the population (i.e. providing sufficient calories per person), food system policies should focus on “nourishing” the population (Searchinger et al., 2019) – with an emphasis on healthy and sufficient food for all from food systems. The food system consists of all the relevant actors (environment, inputs, people, infrastructures, institutions and etc.) along with the activities that relate to the production, processing, distribution, preparation and consumption of food, and the associated outputs of these activities, including socio-economic and environmental outcomes (HLPE, 2014).

A food system framework presents pathways in which a set of complex drivers of change affect the food system including unforeseen consequences acting together and feeding into the system to shape its outcomes (Béné et al., 2019). Data on different components of the food system framework (HLPE, 2020), including on political and economic drivers, innovation, technology and infrastructure, demographic drivers and food supply can help in understanding the specific interrelationships that act together to shape food system outcomes but are currently underutilized.

The continuous collection of reliable (national) data on a large spectrum of food system indicators is crucial for evidence-based decision-making aimed at improving nutritional health in Africa (Annan, 2018). However, the availability of reliable country-level data on the state of food systems to inform policy has been a consistent challenge in West Africa – partly related to limited resources (Annan, 2018). Recent initiatives, including the Food Systems Dashboard (Fanzo et al., 2020), the Global Dietary Database (Global Dietary Database, 2022), and the Global Nutrition Report (Development Initiatives, 2021a) could provide a suitable alternative to access national level data on food system drivers and outcomes. These are useful initiatives but often operate more at a global level and a single “one size fits all” solution is unlikely to apply well in different country-specific contexts. More tailored analyses focusing on national level statistics have added advantages – allowing for the inclusion of context specific food system indicators such as remittances that are important sources of income for rural households in The Gambia (Gambia Bureau of Statistics, 2017). Country-specific analyses are also a means to test the local applicability of indicators included in global databases.

Therefore, this descriptive study sought to bring together data from different open-access global databases on political and economic drivers, demographic drivers, supply chains and nutrition and health outcomes between 1990 and 2017 to study potential leverage points for improvement in the Gambian food system. It compares trends in food supply and nutritional outcomes in The Gambia against West Africa and global averages, and identifies potential drivers based on a food system framework. We demonstrate how useful this kind of analysis could be to identify key priorities for food system transformations using The Gambia as a case-study.

## 2 Methods

### 2.1 Assessment of food system components

In this national food system analysis, we used different open-source databases which provide data on key components of the food systems framework (HLPE, 2017, 2020) for the period between 1990 and 2017. The High Level Panel of Experts (HLPE) on Food Security and Nutrition proposed five key categories of food system drivers that interrelate to influence nutrition and diets (HLPE, 2017). Due to data limitations, our analysis includes data on three drivers: political and economic drivers, innovation, technology and infrastructural drivers, and demographic drivers of food systems change. Other related components of the food systems framework were also analyzed including food supply chains, diets, and nutrition and health outcomes. Table 1 presents the set of food system drivers, intermediaries and outcomes analyzed for The Gambia. We have presented a simple overview of the potential interrelationships among these factors in shaping food system outcomes (specifically: iron deficiency, vitamin A deficiency, and obesity) in The Gambia guided by a Directed Acyclic Graph (DAG) (Greenland et al., 1999) (Supplementary Fig. S1). For example, a possible link between food supply and vitamin A deficiency is likely mediated proximally through the amount of vitamin A rich food supplied from both imports and domestic production (crop yields) (Low et al., 2017). In addition, vitamin A deficiency can also, more distally be a result of increased food purchasing power, for example through employment in agriculture, remittances and GDP. Similarly, living in urban settlements may increase the access of consumers to calorie dense street food – this combined with other urban behavior, such as sedentariness could form important drivers of high obesity prevalence (Bloem and de Pee, 2017) (Supplementary Fig. S1).

**Table 1:** Distribution of selected factors among components of the food system framework.

Drivers of food systems change	Relevant factor(s) analyzed
Political and economic	Gross domestic product, remittances, trade/food importation
Demographic	Urbanization, employment in agriculture
Innovation, technology and infrastructure	Crop yields*
Biophysical and environmental drivers	Not included (NI)
Socio-cultural drivers	NI
**Other components of the food systems framework**	
Food supply chains	Domestic food production, imported food
Diets	Supply of individual food groups
Nutrition and health outcomes	Iron deficiency, vitamin A deficiency, obesity
Consumer behavior	NI
Food environments	NI

*Used as an outcome of this driver.

NI: Variables not included due to lack of data.

Food Systems frame work as defined by HLPE 2017: https://www.fao.org/3/i7846e/i7846e.pdf.

### 2.2 Databases and cleaning

We obtained data from four open-source global databases that provide data on drivers and outcomes of food system change relevant to The Gambia: i) United Nations Food and Agriculture Organization (FAO) Food Balance Sheet (FBS) database (FAO, 2021), ii) World Bank's World Development Indicators database (World Bank, 2021) iii) Global Burden of Disease (GBD) database (Global Burden of Disease Collaborative Network, 2020), and iv) the Non-communicable Diseases (NCD) Risk factor collaboration database (NCD-RisC, 2021).

First, we obtained food supply data from United Nations Food and Agriculture Organization (FAO) Food Balance Sheets (FBS) for the period between 1990 and 2017 (FAO, 2021). These are country level estimates of the amount of food available for human consumption per person per day. FAO Food Balance Sheets data are compiled from a combination of official (government sources such as industrial output surveys, food consumption and expenditure surveys) and unofficial (imputed data) data sources plus its own estimates and data corrections. Details on the methodology behind the compilation of the FAO Food Balance Sheets are given elsewhere (FAO, 2021). Food supply data (average kcal and gram/capita/day) were retrieved for The Gambia, West Africa and globally in order to make comparisons of temporal trends and identify potential related factors. The estimated quantity of food supply for each food item represents the amount available for domestic human consumption by a country's resident population at retail level: comprising production and imports adjusted for exports, stock variation, food losses, food used for seed and animal feed. Food supply data for 97 FAO defined food items were used in the current analysis. These were grouped into six food group aggregates based on shared nutrient content (Willett et al., 2019): (i) cereals and roots, (ii) oils, (iii) animal source foods, (iv) pulses and nuts, (v) fruit and vegetables, and (vi) sugar and sweets. Supplementary Table S2 provides details of the specific food items that make up the food groups. We assessed the adequacy of total energy and food group supply (in grams per person per day) by comparing with the EAT-Lancet recommendations for healthy and sustainable diets (Willett et al., 2019) that have been shown to reflect micronutrient adequacy of diets in low- and middle-income settings (Hanley-Cook et al., 2021).

Furthermore, we obtained annual cereal and vegetable yields from the FAO database for the period 1990–2017. In addition, food production data from the same FAO database, comprising imports and exports of the 97 food items for The Gambia, West Africa and globally between 1990 and 2017 were retrieved. Import dependency ratios (IDRs) by food group for each country were calculated using the following formula (FAO, 2011):

$$IDR=\frac{Import}{Production+Import-Export}$$

Second, we used the World Bank's World Development Indicators database (WDI) (World Bank, 2021) to retrieve country level and global data on annual proportion of people living in urban areas, employment in agriculture, and gross domestic product (GDP) – corrected for country level purchasing power parity for the period 1990 to 2017. Data on total remittances inflow per year were obtained from the World Bank's KNOMAD (https://www.knomad.org/data/remittances). We computed per capita remittances using United Nations population data (FAO, 2019). Remittances per capita were further corrected for variations in purchasing power in different countries by applying World Bank's Purchasing Power Parities (PPP) conversion factors (World Bank, 2021).

Third, age-standardized prevalence data for two nutritional outcomes were obtained from the Global Burden of Disease (GBD) database (Global Burden of Disease Collaborative Network, 2020). Data on age-standardized prevalence of dietary iron deficiency and vitamin A deficiency for country level, regional and globally for the years 1990–2017 (Global Burden of Disease Collaborative Network, 2020) were extracted for this study. Dietary iron and vitamin A deficiency are associated with increased burden of disease and disability in The Gambia and globally (Global Burden of Disease Collaborative Network, 2020) and are more likely driven by food system factors hence their selection as outcomes for the Gambian food system (Beal et al., 2017; National Nutrition Agency-Gambia, 2019). Detailed explanations of how these estimates are derived are provided elsewhere by the GBD (James et al., 2018).

Finally, we used data from the Non-communicable Diseases (NCD) Risk factor collaboration database (NCD-RisC: https://ncdrisc.org/index.html) to derive the age-standardized prevalence of an additional food system outcome variable that is of increasing concern for causing death and disability in The Gambia and globally: high body mass index (obesity) (Murray et al., 2020).

### 2.3 Statistical analysis

We have mainly explored relationships among variables in this study using graphical methods to display and compare trends in different food system indicators due to the aggregate nature of the data. While some databases covered a few more recent years, at the time of data compilation the FAO database reported up to 2017, and hence this was taken as final year of all analyses. Data on remittance inflows were available starting in 2002 for The Gambia and so this was used as the starting point for analysis involving remittances.

We have presented 3-year rolling averages for most estimates to overcome potential reporting inaccuracies associated with reporting yearly data and to account for potential time lags between food supply and outcomes. Nutritional outcome variables were selected based on country-specific prevalence and associated disease burden and include: iron deficiency, vitamin A deficiency (Beal et al., 2017; National Nutrition Agency-Gambia, 2019) and obesity (Cham et al., 2020; Murray et al., 2020). We performed pairwise correlations, with Bonferroni corrections to explore interrelationships between the economic and demographic factors (GDP, remittances, urbanization and employment in agriculture) and also their association with the selected outcomes. We also graphically analyzed intermediate relationships between the economic and demographic factors and the supply of total energy and specific food groups. Finally, we present graphical relationships between food groups supply the selected outcomes in The Gambia.

## 3 Results

### 3.1 Trends in GDP, remittances, urbanization and employment in agriculture in the Gambia

**Fig. 1:**
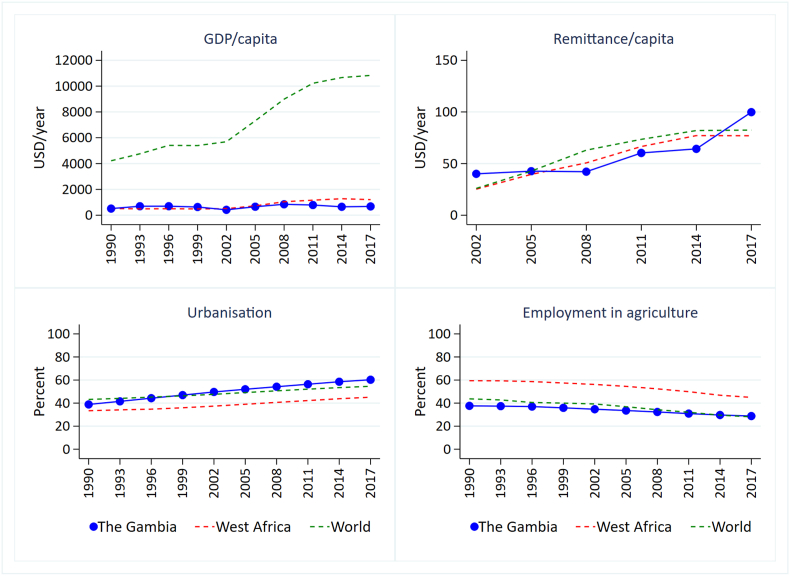
GDP, remittances, urbanization and agriculture employment transitions in The Gambia, West Africa and globally 1990 to 2017.

The Gambia's GDP has increased only slightly since 1990. Urbanization has increased rapidly such that over 60% of the population now live in urban areas. Remittances from overseas have more than doubled, while employment in agriculture has decreased (Fig. 1).

Compared with the wider West African region, per capita remittances rose slowly in The Gambia until 2015 when they rose higher than both West African and global averages. However, remittances had significantly lower purchasing value in The Gambia than in other West African countries when corrected for country level parities in purchasing power (Supplementary Fig. S2). Therefore, an equal amount of remittances received in The Gambia can purchase less goods and services than it would do elsewhere in West Africa. Rates of urbanization in The Gambia are higher than in West Africa on average and employment in agriculture is lower. Employment in agriculture in The Gambia is broadly similar to the global average but substantially lower than other West African countries and still decreasing (Fig. 1).

### 3.2 Changes in food supply

Since 2005 the average daily supply of energy per person has been higher than the 2503 kcal (Supplementary Fig. S3) specified by EAT-Lancet dietary recommendations. Recent data on food prices show minimal year-round variations in market prices of major food groups – prices of animal source foods were the most stable throughout the year (Supplementary Table S3).

**Fig. 2:**
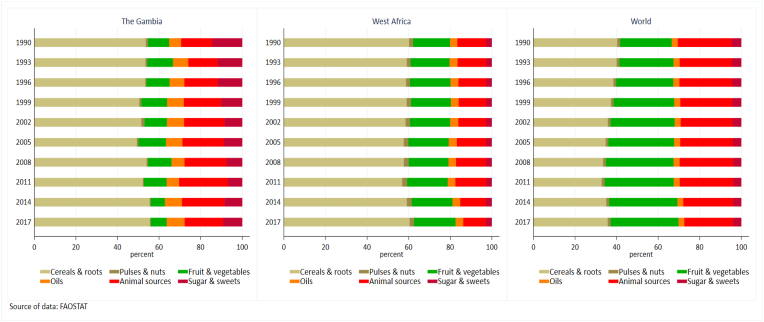
Transitions in food supply in The Gambia, West Africa and globally 1990 to 2017 [Food supply is in gram/capita/day].

In terms of dietary composition however, average diets in The Gambia appear to have low diversity, with cereals and roots making up the largest part of the diet (54% in 1990 and 56% in 2017; Fig. 2). This has not significantly improved over the past decades. Furthermore, fruit and vegetable availability is extremely low (with a steady declining trend over time from 89 to 68g/person/d between 1990 and 2017), while supply of animal source foods has – on average – been stable (∼16% of the diet). The supply of sugars and sweets also declined steadily (from 15% in 1990 to 9% 2017) (Fig. 2).

Compared to the EAT-Lancet diet recommendations, amounts of cereals available in The Gambia are comparable to the upper limit of the recommended amounts for consumption by the EAT-Lancet (EAT-Lancet recommendation:464g; average per capita supply in The Gambia: 495g in 2017) compared to very high per capita supply of cereals in both West Africa (839g in 2017) and globally (649g in 2017). Fruit and vegetable supply in The Gambia is substantially lower than the West African average, and is currently estimated at 68g/person/d in 2017 compared to 276g/person/d in the region, both being below the 500g/person/day EAT-Lancet recommendation – but globally, the supply of fruit and vegetables were higher (592g/person/d in 2017).

The supply of animal source foods is below the EAT-Lancet maximum recommendation of 334g/person/d (meat, poultry, fish, dairy and eggs) for The Gambia (164g/person/d in 2017) and West Africa (151g/person/d in 2017), in contrast to the global average which is well above this recommendation at 428g/person/d in 2017. The supply of pulses and nuts was particularly low in The Gambia (9g/person/d in 1990 to 4g/person/d in 2017) compared to West Africa (22g in 1990 to 30g in 2017) and the global average (20g/person/d in 1990 to 25g/person/d in 2017) and well below the EAT-Lancet recommendation of 125g per person per day (Supplementary Table S2).

In contrast, the supply of oils and sugars is higher in The Gambia compared to both West African and global averages (Fig. 2 and Supplementary Table S2). Supply of sugar in The Gambia is higher than the 31g/person/day maximum intake level recommended by the EAT-Lancet diet despite being reduced from 128g/person/d in 1990 to 82g/person/d in 2017. Comparatively, the supply of sugar in West Africa and the global average were 40g/person/d and 72g/person/d respectively in 2017. The average supply of oils and fats in The Gambia is also higher (77g/person/d in 2017) than the West African average (52g/person/d) and the global average (51g/person/d in 2017) but are all within the EAT-Lancet recommendation of no more than 92g/person/d (Fig. 2 and Supplementary Table S2).

### 3.3 Trends in food production and trade

**Fig. 3:**
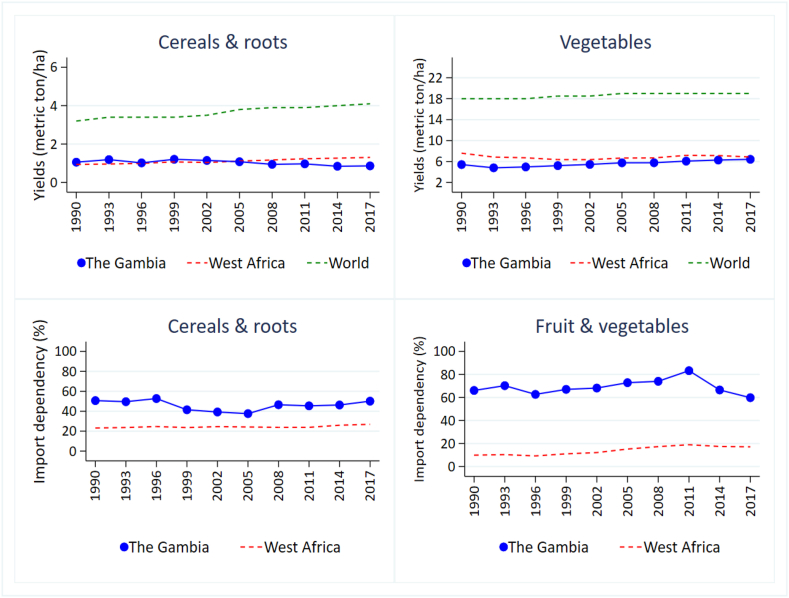
Yields and imports of cereals and vegetables in The Gambia, West Africa and globally 1990 to 2017.

To further explore driving factors that may be associated with low availability of some foods in The Gambia, we investigated trends in yields and imports for both the cereal crops providing the bulk of dietary energy and nutritionally important fruits and vegetables (Fig. 3).

Yields of both cereals and vegetables in The Gambia are low compared to the global mean, averaging below 2 metric tonnes per hectare for cereals and around 5 metric tonnes per hectare for vegetables (Estimate excludes fruit yields) between 1990 and 2017. Dependence on imports of fruit and vegetables (>60%) and cereals (>50%) from other countries is high compared to West African averages. In some years, the proportion of fruit and vegetables imported in The Gambia has reached 80%, indicating very low levels of domestic production. Increasing cereal and vegetable yields were related to the dependence on imports over time. For example, in 2011, there was an inverse relationship where imports start declining and local production increases (Supplementary Fig. S4).

### 3.4 Changes in nutrition and health outcomes in The Gambia

**Fig. 4:**
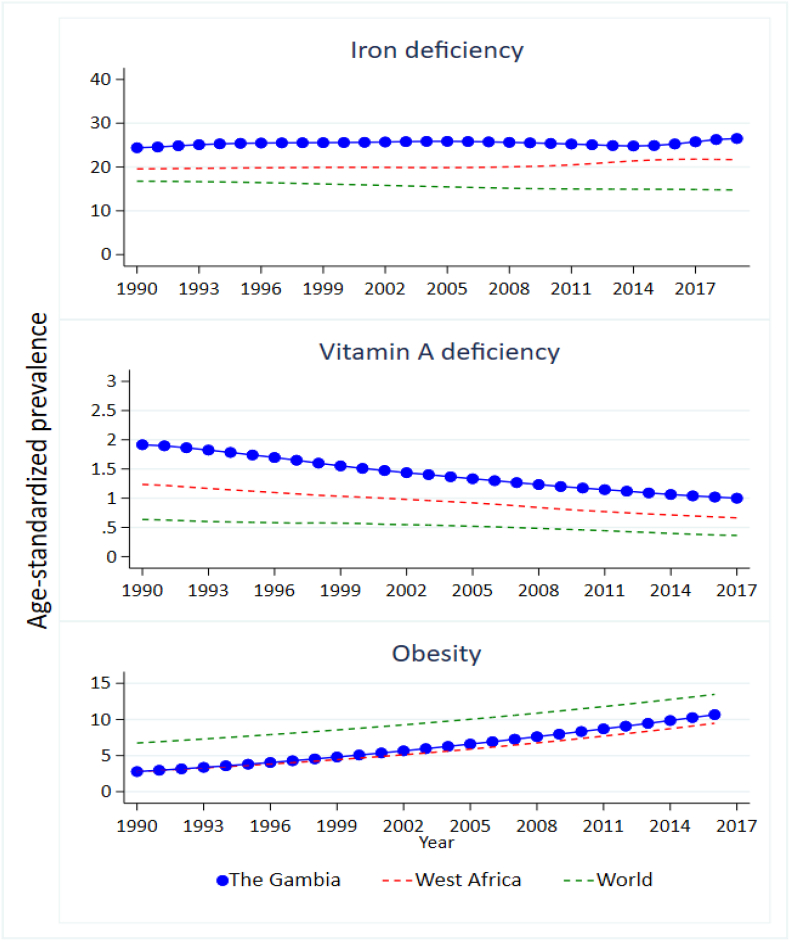
Nutrition and health transitions in The Gambia, West Africa and globally 1990 to 2017.

Over the past decades, iron deficiency rates in The Gambia have been substantially higher than the West African average (Fig. 4). Between 1990 and 2017, the age-adjusted prevalence of iron deficiency increased from 24.4% to 26.5%. The age-adjusted prevalence of vitamin A deficiency has been low with a current downward trend, but remains higher than the West African average. The reported prevalence of vitamin A deficiency of 2.0% in 1990 had halved (1.0%) in 2017.

At the same time an increase is noticeable in the proportion of adults classified as obese (10.7% in 2016 from 2.8% in 1990). At a global scale, the prevalence in Gambia is still relatively low, as the global obesity prevalence is currently 13.5%, but in the past 20 years prevalence in The Gambia has increased to be greater than the West African average (Fig. 4).

### 3.5 Trends in food supply and nutrition and health outcomes in The Gambia

Exploratory correlation analysis of interrelationships among the political and economic as well as demographic drivers, and nutritional outcomes showed that greater employment in agriculture was associated with lower levels of obesity, but was also associated with high levels of vitamin A deficiency (Supplementary Table S4).

Greater levels of urbanization were associated with reduced employment in agriculture and higher levels of obesity, but did not show a significant relationship with other nutritional outcomes.

Receipt of remittances was significantly correlated with a number of other drivers: increased remittances were associated with greater urbanization and reduced employment in agriculture, and with increased prevalence of obesity. However, they were also associated with lower prevalence of vitamin A deficiency. Growth in GDP, conversely, showed no significant correlations with other drivers or with nutritional outcomes.

In terms of crop yields, higher cereal yields were related to greater employment in agriculture but were also associated with higher levels of vitamin A deficiency, while higher vegetable yields were associated with lower vitamin A deficiency. Crop yields were not significantly related to levels of iron deficiency (Supplementary Table S4).

**Fig. 5:**
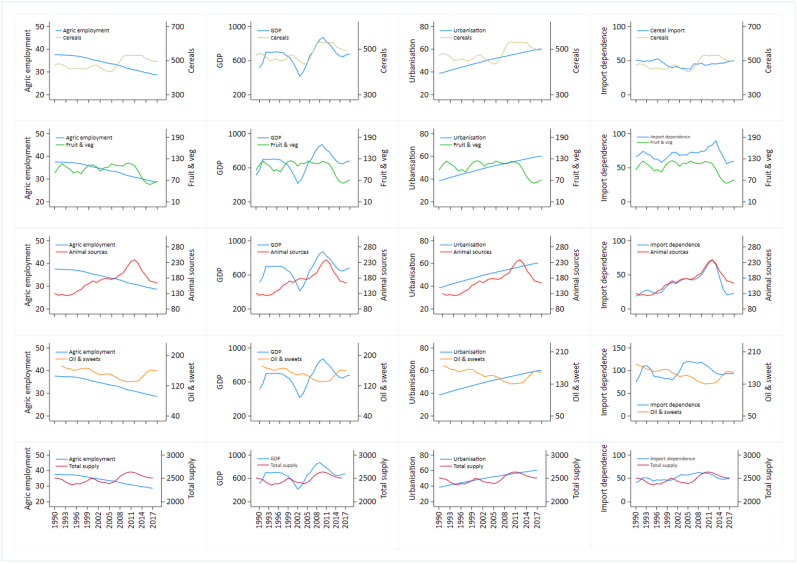
Potential predictors of food supply in The Gambia (1990–2017) [The specific food groups are in g/person/day; total supply is in kcal/person/day; GDP is in USD/year; employment in agriculture is in percent of total employment/year; urbanization is in percent/year; import dependence is in percent/year].

Graphical exploration indicated that over time in The Gambia (notwithstanding the fact that these relations cannot be assumed to be causal), increases in both GDP and urbanization both tended to pre-date increases in the supply of cereals, animal source food, oils, and total food. Remittances did not appear related to the availability of specific food groups directly. Food import was a key factor for the availability of all food groups in The Gambia with over 50% of total supply coming from food import. Importantly, economic factors did not appear related to increased availability of fruit and vegetables (Fig. 5 and Supplementary Fig. S5).

Finally, we explore graphical associations between availability of specific food groups (and energy) and nutrition and health outcomes.^1^ An increase in the supply of animal source foods tended to pre-date a decrease in iron deficiency, and an increase in the availability of oils also tended to pre-date changes in vitamin A deficiency while an increase in the overall food availability appeared to pre-date reductions in both iron and vitamin A deficiency. Obesity related only weakly with supply of oils and total food availability in the country (Supplementary Fig. S6).

## 4 Discussion

### 4.1 Main findings

Large, open-source routinely-collected datasets are a useful tool to explore current national food systems, their trends and their comparison in regional and global contexts, especially in settings with limited availability of country specific data. Our analyses show that, over the past three decades in The Gambia, supply of specific food groups and total energy have increased (even though iron deficiency is still a problem) and overall diet composition is poor with less fruit and vegetables, and more sugar and oils compared to neighbouring countries and global supply averages. Obesity prevalence is rising quickly possibly related to increased urbanization and less engagement in agricultural work. Furthermore, domestic production is low throughout all food groups. High imports of cereals, animal source foods and sugar from other countries seem to bridge the gap in terms of energy supply, but low imports of fruit and vegetables in combination with low domestic supply overall may be leading to micronutrient deficiencies. GDP and urbanization appear to predict the supply of specific food groups in The Gambia, but there is a lack of relationship with employment in agriculture which may reflect the country's persisting low levels of domestic production and crop yields. With ongoing urbanization rates outstripping regional and global levels along with continually declining crop yields and levels of agricultural employment, the trends in food supply may worsen in the future without extensive reliance on imports.

### 4.2 Research in context

The greater reduction in agricultural employment in The Gambia compared to other West African countries may indicate a more prevalent diversification of livelihoods from agriculture in The Gambia, and may also be linked with burgeoning tourism in the country in recent years (Mitchell and Faal, 2007). The lower crop yields may indicate significant production inefficiency in the Gambian agriculture sector and shows the potential for increased domestic supply despite the low numbers employed in the sector. Employment in agriculture did not appear to relate to changes in availability of any food groups even though it was strongly related to crop yields, which may reflect the high degree of dependence on imports in the Gambian food system. For instance, increasing cereal and vegetable yields were related to the dependence on imports over time. This implies that increasing domestic production efficiency would have potential to cut the reliance on imports in the country. Further, we show that selected political and economic drivers and demographic drivers did not appear related to the availability of nutritionally important fruit and vegetables. This may mean that without targeted efforts to increase their supply, usual economic growth is unlikely to lead to an increase in the availability of fruit and vegetables.

The increasing supply of energy which reached adequate levels (compared to EAT-Lancet targets) in 2005 could mean sufficient food energy and may partly explain the corresponding decline in prevalence of undernourishment (FAO et al., 2021). Given that inequitable food distributions are highly likely and that the data do not account for food wastage, the small margin above the recommended intake level may also indicate food insecurity for parts of the population (Mangan, 2021). In contrast, rising obesity also shows that sections of the population still have excess energy supply, presenting a problem with its own associated health and economic burdens (Chu et al., 2018).

There is growing realisation of the importance of national level transitions such as urbanization and economic growth on obesity (Ruel et al., 2017). In The Gambia, urban settlement has traditionally been high and has continued to increase (World Bank, 2021). Hence, the rather recent steep rises in obesity rates can only be partly explained by continuing urbanization and highlighting the role of energy dense food groups such as vegetable oil and sugar. Rising obesity levels in low-and middle-income countries are thought to play through both globalization (the flooding of low-income country markets with inexpensive and high caloric foods) and modernization which recognizes domestic factors (such as an increase in intake of unhealthy foods in response to rising income, automation of processes and increased retail outlets which reduce distance to markets) (Fox et al., 2019). The latter is the most likely culprit, but both concepts have likely played a role in The Gambia's obesity epidemic (Cham et al., 2020; Webb and Prentice, 2006).

Growing urbanization and declines in crop yields over time may be due to movement to urban areas and neglect of crop production in rural areas. This can also be due to loss of incentives for farm work following income from remittances (mainly from diaspora family members (Gambia Bureau of Statistics, 2017)) coupled with a lack of profit from declining crop yields. The yield gap in The Gambia is the result of a combination of factors including low soil fertility from low fertilizer use, poor cultivation practices and climate uncertainties (drought, irregular rainfall pattern and salt intrusion of crop land) (FAO et al., 2018). Availability of cheap imported cereals (mainly rice, maize and wheat flour) and consecutive crop production failures (Bagagnan et al., 2019; Sonko et al., 2020) have also led to a reliance on imported food rather than investment in domestic production. The relationship between GDP growth and cereal supply could therefore be explained by increased food importation rather than boosting domestic production through the provision of farm inputs and mechanization.

International food trade including bilateral donations of food can be an efficient way to complement domestic supply insufficiency (Kummu et al., 2020). But over reliance on food imports or aid, especially if such crops are the main staple food, can increase the vulnerability of food supply in consuming countries to shocks such as climate change and crop failure in food producing countries. Food imports and food donations have, so far, successfully bridged the gap in domestic production for most food groups, particularly: cereals, animal sources, oils and sugar. Reliance on rice as a main staple food seems to drive the overall dependence on cereal imports in the country. This is similar to neighbouring countries such as Niger and Senegal where rice is a major staple (Fontan Sers and Mughal, 2020). In these rice dominated countries, there is low domestic production due in part to consumer preference for polished imported rice (Demont et al., 2017). In contrast, in less rice reliant countries with a diverse number of staple foods, dependency on cereal imports is far lower. For instance, in Nigeria and Ghana (the two most populated countries in the region) where maize, cassava and yams are major national staples (Ekpa et al., 2018; Haleegoah et al., 2015; Olayiwola et al., 2012) (mainly produced and processed locally), the dependency on cereal imports is below 10%.

Unlike cereals and other food groups, the gap in domestic supply of fruit and vegetables has not been met through imports and hence there is low overall supply. While this is a consistent trend with regional supply levels, actual amounts available per person in The Gambia are far lower and are inadequate when compared with amounts set by WHO to meet health needs (WHO, 2003) and the sustainable diet target by EAT-Lancet (Willett et al., 2019). There is little documentation on informal cross-border trade of fruit and vegetables within the West African region despite recent efforts (CILSS, 2021). However, a recent study that performed a comprehensive country-of-origin tracing of fruit and vegetables imports showed that between 1988 and 2018, The Gambia largely imported fruit and vegetables from climate stable countries outside the West Africa region (Hadida et al., 2022). The low supply of fruit and vegetables is consistent with evidence from a national survey which showed that only 7% of adults aged 25–64 years consumed five servings of fruit and vegetables per day in The Gambia (Government of Gambia, 2010). The majority of the agricultural workforce in The Gambia is engaged in horticultural production (65% of all employed in agriculture) (United Purpose, 2018). In spite of this, domestic production meets only 18% of national demand. A significant proportion of this amount goes into hotel and restaurant chains and is consumed by tourists and wealthy local residents, further reducing the amounts available for the wider public (United Purpose, 2018). With similar trends in vegetable yields compared to regional averages, the low domestic supply is possibly due to high post harvest losses or less cultivated land. There are also large seasonal fluctuations in availability of fruit and vegetables in the country with gluts of mangoes, oranges and watermelons concentrated within short periods (Bates et al., 1982; United Purpose, 2018). These are associated with major fluctuations in vitamin C status for instance (Bates et al., 1982). Therefore, without proper irrigation farming (currently underutilized) (Segnon et al., 2021), improved storage facilities and control of post-harvest losses, dependence on imports from other countries seems to be a more suitable way to ensure consistent supply of fruit and vegetables throughout the year.

### 4.3 Strengths and limitations

This study has demonstrated the utility of combining food system and economic development data to understand a country's food system situation with reference to regional and global contexts. It brings further use to routinely collected and often expensive data by governments and development partners which individual surveys will be unable to achieve independently.

However, this study has several limitations worth noting when interpreting the meanings of these results. Chiefly, the data used for the analysis are ecological in nature and therefore causality cannot be determined, only inferred from the kinds of graphical relationships presented here showing the onset of different trends over time. We were also unable to explore differences among population groups in The Gambia in terms of food consumption or income levels etc., and could only rely on national averages. These kinds of national data should be supplemented by additional sources such as national household or dietary surveys in order to give a more complete picture of individual food systems. Our analysis of food supply was limited to production, imports and overall availability and did not consider other important factors such as changes in markets, trade, storage and affordability. The food availability analysis did not also account for bioavailability, enhancers, and inhibitors as well as fortification of specific foods which can affect deficiency dynamics independent of food supply levels. For example, the relationship between supply of oils and vitamin A deficiency is likely the result of fortification of vegetable oil with vitamin A. The use of EAT-Lancet dietary guidelines was also limited to adult diets and not children, pregnant and lactating women who have heightened nutrient needs and are very vulnerable to nutrient deficiency in this population (National Nutrition Agency-Gambia, 2019).

Reliance on data from secondary sources also implies that the trends presented are only as accurate as the original surveys or any further data processing performed by database holders. Of particular note is the FAO Food Balance Sheet data that have been criticised for not reflecting actual consumption with major overestimations for high income countries while underestimating consumption in low-income settings (Del Gobbo et al., 2015). For example, onions are the largest produced vegetables in The Gambia (United Purpose, 2018), yet these are not included in FAO's calculation of vegetable supply for The Gambia (FAO, 2021) leading to an underestimation of overall availability of vegetables in the country. The trends in supply of vegetables over time may however be important as the set of included vegetables is reported consistently. Further, the FAO food supply data do not account for small-scale production and wild crops that are likely to constitute a significant part of rural diets. This may imply that our analysis of food supply is likely more relevant to urban populations than rural dwellers even though availability of imported foods such as rice, wheat (flour and bread), sugar, oils, tomato paste etc (included in FAO statistics) are high in rural areas (Gambia Bureau of Statistics, 2016). The limitations of data and sampling methods of the other databases used in this study are detailed elsewhere (James et al., 2018; NCD-RisC, 2021; World Bank, 2014).

### 4.4 Recommendations

From the 2008 global economic crisis that impacted heavily on prices of imported food and ongoing impacts of climate change (Epule et al., 2014), the government of Gambia's current agricultural policy aims to increase domestic production and achieve self-sufficiency by 2026 (Government of Gambia, 2019). While this is commendable, there should be consideration of the realistic proportion of food that can be produced domestically given the resources available. A recent global analysis showed that stability in national dietary diversity can be improved through crop diversification with strong contributions from imported food (Nicholson et al., 2021). Therefore, a certain level of food import will be important to ensure improved national diet diversity and supply sufficiency. This should be done through comprehensive evidence mapping of the economic and environmental trade offs that come with increasing domestic production and import sources. Diversification in cereals used as main staple food should be considered to reduce reliance on rice. Promoting the production and consumption of biofortified crops such as pearl millet, maize, and cassava as alternative staple foods in the country could be a viable strategy for diversification and combat micronutrient deficiency, as availability and consumption of these is increasing (FAO-Gambia, 2020). The diversification of food import sources could also be a strategy to reduce supply vulnerability such that disruptions in one producing country will not impact heavily on the food supply.

To meet fruit and vegetable supply deficits, substantial increases in current amounts from both domestic supply and imports are needed. This can be done by taking advantage of the large labour force working in horticultural production in the country – including in urban areas (CTA, 1991). The promotion of small-urban vegetable production may have additional benefits to effectively complement rural production through shorter supply chains (FAO, 2007). It may lead to less sedentary lifestyles (Cham et al., 2020) and serve as a ‘double duty’ intervention to address both vegetable supply problems and increase physical activity. Increasing urbanization rates may also release large pieces of land in rural areas which could be harnessed for large scale mechanized and sustainable intensification agriculture that is shown to improve domestic food supply sufficiency (Wang et al., 2021). There is also the need to improve assessment of fruit and vegetable production in the country by making them a major component of routine national surveys such as the National Agricultural Survey (NAS). This will improve understanding of actual supply and improve the quality of data hosted in global databases including FAOSTAT, Food System Dashboard and the Global Dietary Database.

The variables considered in this analysis constitute a small aspect of the food system, as many different components of the food system framework that interrelate to shape food system outcomes such as biophysical and environmental drivers, technology and innovation, food environments and socio-cultural factors (HLPE, 2020) as well as different dynamics of food security such as food access and utilization were not included. These should be considered in future studies of food system analysis for a more holistic understanding of food system outcomes.

## 5 Conclusion

Open-source routinely collected global data is a useful tool for food system analyses, especially in settings with limited data collection and availability at national level, such as The Gambia. We can conclude that measures of development, particularly GDP, were positively related to supply of cereals and animal source foods over time, but no such relationship was observed for fruit and vegetable supply. Food system policy to improve nutrition and health outcomes in The Gambia needs to focus on improving the diversity of food supply and imports – especially fruit and vegetables – and maximize domestic production to reduce reliance on food import.

## Declaration of competing interest

The authors declare that they have no known competing financial interests or personal relationships that could have appeared to influence the work reported in this paper.

## Data Availability

Data will be made available on request.

## Footnotes

1 Only a selection of food groups with a plausible nutritional link with outcome variables were explored.
