# Supplementary material for: Socio-economic and food system drivers of nutrition and health transitions in The Gambia from 1990 to 2017
Source: Glob Food Sec. 2023 Jun;37:100695. doi: 10.1016/j.gfs.2023.100695 (PMC10334500; doi:10.1016/j.gfs.2023.100695)
Supplement: Multimedia component 1 [file mmc1.docx]

# Supplementary material


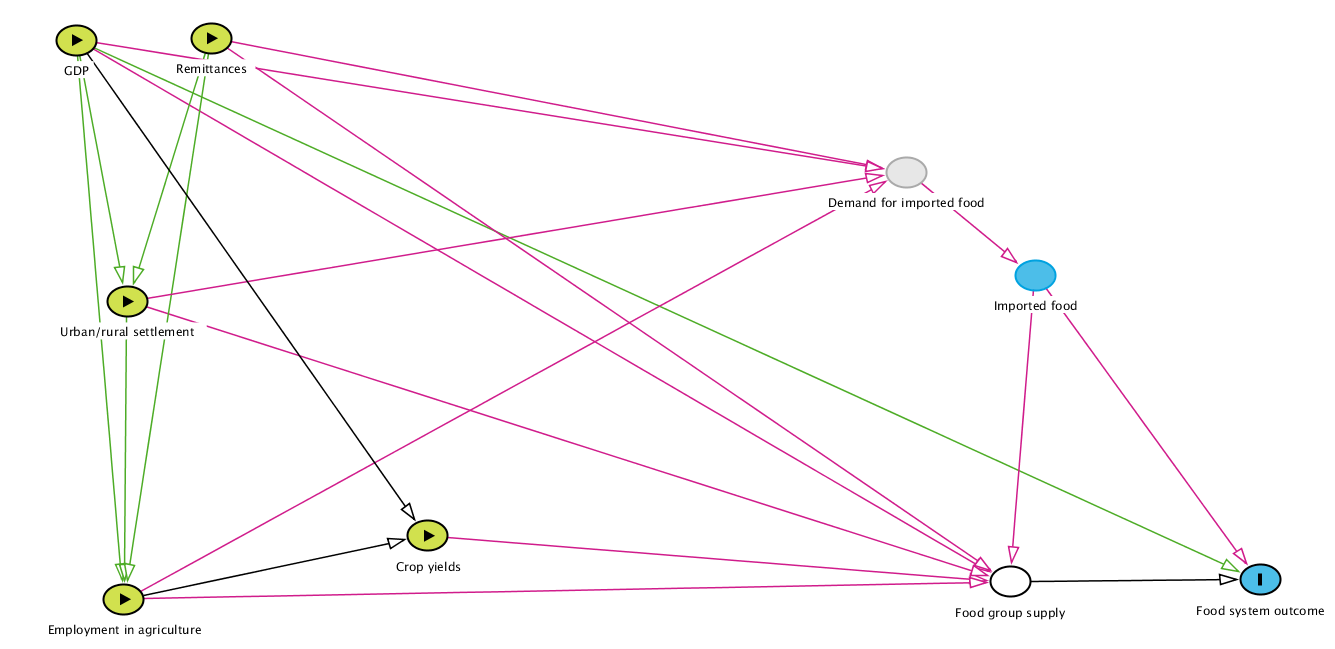
**Figure S1**: Theorized pathways of the structural determinants of nutrition and health outcomes in The Gambi

**Table S1**: Food groups and constituent food items

| Food groups* | Cereals and roots | Oils | Animal sources | Pulses and nuts | Fruit and vegetables | Sweets and sugar |
| --- | --- | --- | --- | --- | --- | --- |
| Constituent food items | Wheat, rice, barley, maize, rye, oats, millet, sorghum, other cereal, potato, cassava, sweet potato, other root, yam | Fish liver oil, fish body oil, cream, butter, raw animal fat, other oil, maize germ oil, rice bran oil, olive oil, sesame seed oil, coconut oil, palm oil, palm kernel oil, cotton seed oil, mustard/rapeseed oil, sunflower oil, groundnut oil, soyabean oil | Bovine meat, mutton, pig meat, poultry, other meat, edible offal, eggs, fresh water fish, demersal fish, pelagic fish, other marine fish, crustaceans, cephalopods, molluscs, aquatic mammal, other aquatic animal, milk. | Beans, peas, other pulse, nuts, soyabean, groundnuts, sunflower seed, rapeseed/mustard, cotton seed, coconut, sesame seed, palm kernel, other oil crop, cocoa beans and products. | Tomatoes, onions, other vegetable, orange/mandarins, lemon/lime, grape fruit, other citrus, banana, plantain, apple, pineapple, dates, grape product, other fruit, pepper, pimento, cloves | sugar cane, sugar beet, non-centrifugal sugar, raw sugar, honey, other sweetener |
| Total | 14 | 18 | 17 | 14 | 17 | 6 |

Food constituents follow FAO official groupings and where other is stated, constituents are often a cluster of many more food items defined by FAO. For example, other vegetables contain: Cabbages and other brassicas, Artichokes, Asparagus, Lettuce and chicory, Spinach, Cassava leaves, Cauliflowers and broccoli, Pumpkins, squash and gourds, Cucumbers and gherkins, Eggplants (aubergines), Chillies and peppers, green, Onions, shallots, green, Garlic, Leeks, other alliaceous vegetables, Beans, green, Peas, green, Vegetables, leguminous nes, String beans, Carrots and turnips, Okra, Maize, green, Sweet corn frozen, Sweet corn prep or preserved, Mushrooms and truffles, Mushrooms, dried, Mushrooms, canned, Chicory roots, Carobs, Vegetables, fresh nes, Vegetables, dried nes, Vegetables, canned nes, Juice, vegetables nes, Vegetables, dehydrated, Vegetables in vinegar, Vegetables, preserved nes, Vegetables, frozen, Vegetables, temporarily preserved, Vegetables, preserved, frozen, Vegetables, homogenized preparations, Watermelons, Melons, other (inc.cantaloupes), Coffee, substitutes containing coffee

**Table S2**: EAT-Lancet diet recommendations and food group supply

| EAT-Lancet food group | Target intake (range in grams) | Functional category used in analysis | The Gambia* | | West Africa* | | World* | |
| --- | --- | --- | --- | --- | --- | --- | --- | --- |
|  |  |  | 1990 | 2017 | 1990 | 2017 | 1990 | 2017 |
| Vegetables | 300 (200-600) | Fruit and vegetables | 89.49 | 67.56 | 201.55 | 276.47 | 347.73 | 592.34 |
| Fruits | 200 (100-300) |  |  |  |  |  |  |  |
| Beans, lentils, peas | 75 (0-150) | Pulses and nuts | 9.32 | 4.12 | 21.63 | 29.56 | 19.77 | 25.39 |
| Peanuts and tree nuts | 50 (0-100) |  |  |  |  |  |  |  |
| Cereals and grains | 232 (0-464) | Cereals and roots | 472.74 | 495.38 | 679.31 | 839.16 | 564.97 | 648.98 |
| Potatoes and cassava | 50 (0-100) |  |  |  |  |  |  |  |
| Unsaturated oils | 40 (20-80) | Oils | 51.31 | 77.23 | 39.36 | 51.79 | 40.56 | 50.83 |
| Palm oil | 6.8 (0-6.8) |  |  |  |  |  |  |  |
| Lard or tallow | 5 (0-5) |  |  |  |  |  |  |  |
| Added sugar | 31 (0-31) | Sugars and sweets | 127.67 | 82.08 | 30.59 | 39.51 | 66.32 | 71.90 |
| Fish | 28 (0-100) | Animal source foods | 131.04 | 164.29 | 156.01 | 150.50 | 361.61 | 426.08 |
| Beef and lamb | 7 (0-14) |  |  |  |  |  |  |  |
| Pork | 7 (0-14) |  |  |  |  |  |  |  |
| Poultry | 29 (0-58) |  |  |  |  |  |  |  |
| Dairy | 250 (0-500) |  |  |  |  |  |  |  |
| Eggs | 13 (0-25) |  |  |  |  |  |  |  |

*Food group supply is in gram per capita per day.

**Table S3**: Average monthly market prices of major food commodities in 25 markets in The Gambia 2022 (GMD/kg)

| **Cereals** | **Jan** | **Feb** | **Mar** | **Apr** | **May** | **Jun** | **Jul** | **Aug** | **Sep** | **Oct** | **Nov** | **% change (Jan-Nov)** |
| --- | --- | --- | --- | --- | --- | --- | --- | --- | --- | --- | --- | --- |
| Maize | 29 | 29 | 30 | 31.67 | 31.49 | 37.73 | 38.67 | 38.64 | 33.27 | 37.06 | 38.77 | 33.69 |
| Millet | 29 | 26.86 | 29.75 | 31.33 | 31.49 | 34.22 | 38.14 | 38.71 | 32.45 | 36.97 | 37.91 | 30.72 |
| Sorghum | 29 | 28 | 31 | 33 | 32 | 38.25 | 42 | 42.14 | 34.47 | 39.02 | 43.27 | 49.21 |
| Local rice | 28 | 32.67 | 40 | 35.5 | 38 | 40.33 | 41 | 41 | 37.08 | 40.2 | 37.06 | 32.36 |
| Broken rice | 27 | 31.2 | 32.25 | 33 | 35 | 35.7 | 35.78 | 35.5 | 33.18 | 36.18 | 40.2 | 48.89 |
| Long grain rice | 29 | 34 | 36.25 | 35.67 | 38 | 40 | 40 | 39.82 | 36.69 | 39.36 | 40.1 | 38.28 |
| **Legumes** |  |  |  |  |  |  |  |  |  |  |  |  |
| Decorticated groundnut | 70 | 66.14 | 66.33 | 66 | 65.28 | 86 | 88 | 91.5 | 87.67 | 92.59 | 98.62 | 40.89 |
| Undecorticated groundnut | 63 | 55 | 64 | 67 | 64 | 78.4 | 80.33 | 83.93 | 85.08 | 87.68 | 85 | 34.92 |
| White beans | 79 | 80 | 80.67 | 84 | 87 | 94.75 | 96.25 | 101.77 | 101.8 | 102 | 102 | 29.11 |
| Red beans | 81 | 77.5 | 82.25 | 86.33 | 96.39 | 106.33 | 107.5 | 109.68 | 105 | 104.01 | 105.11 | 29.77 |
| **Livestock products** |  |  |  |  |  |  |  |  |  |  |  |  |
| Meat with bone | 250 | 250 | 250 | 250 | 276.67 | 284.09 | 288.89 | 295.83 | 287.18 | 287.8 | 287.18 | 14.87 |
| Beef steak | 300 | 300 | 300 | 300 | 325 | 334.09 | 338.89 | 339.58 | 332 | 332 | 332.44 | 10.81 |
| Mutton | 350 | 345 | 350 | 350 | 350 | 338.64 | 341.67 | 350 | 346.91 | 346 | 346.91 | -0.88 |

**Source of data:** The Gambia Market Information Bulletin, Volume 7, November 2022. Department of Planning, Ministry of Agriculture. **Note:** 1 USD = 61.15 GMD at time of report in November 2022.


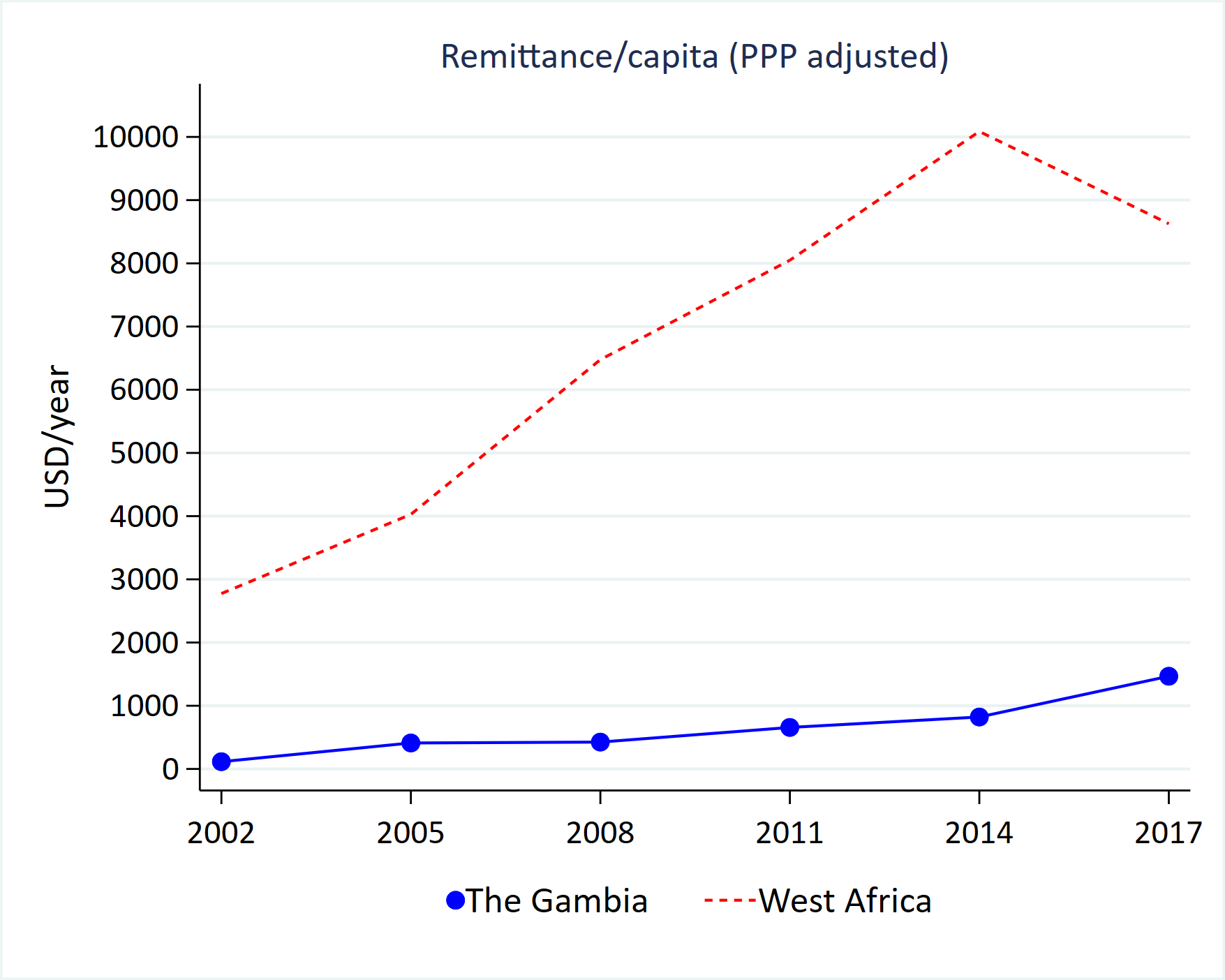


**Figure S2**: Trends in remittances adjusted for country-level purchasing power parities (PPP)


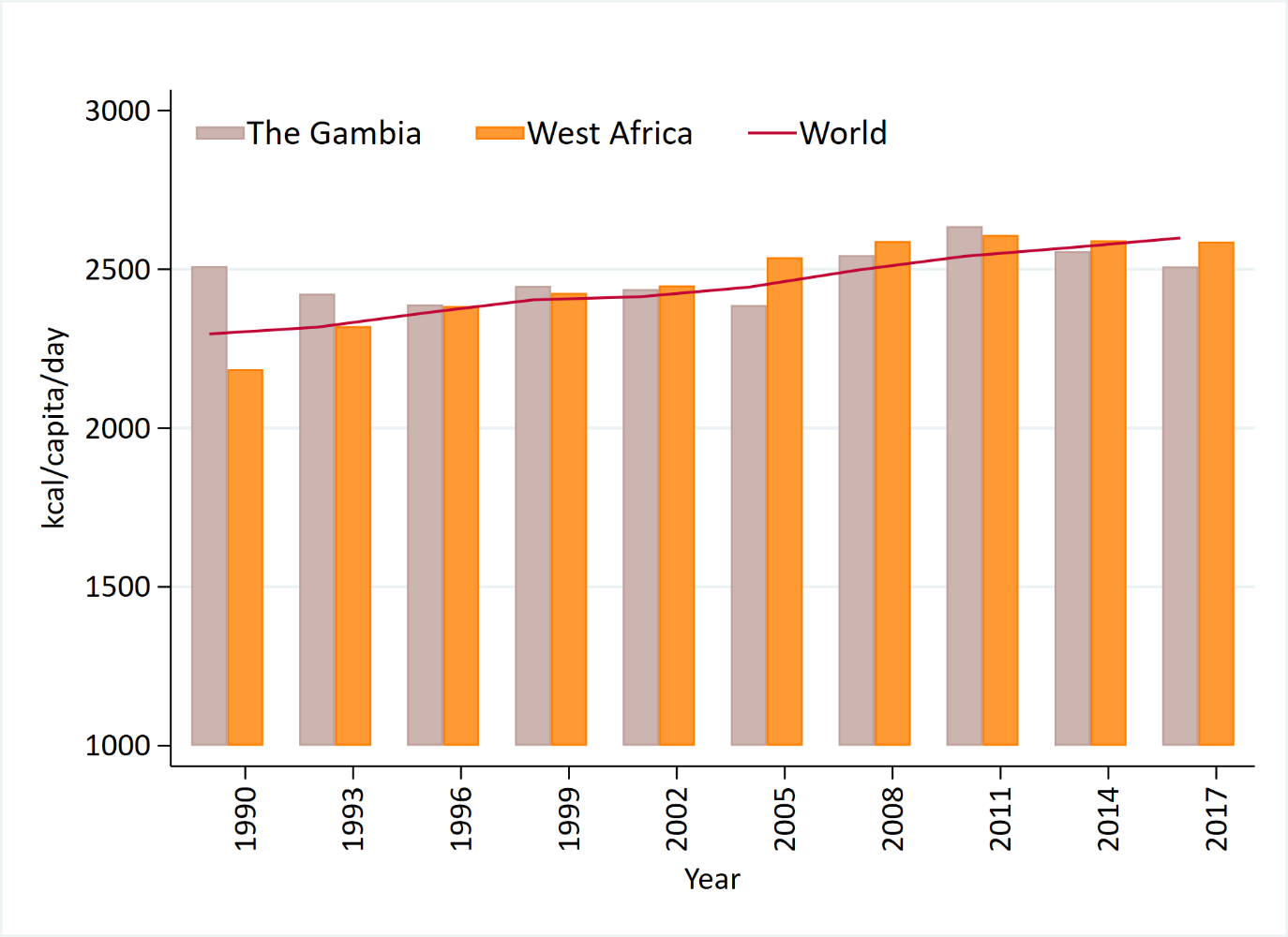


**Figure S3:** Supply of total calories in The Gambia, West Africa and globally (1990-2017)


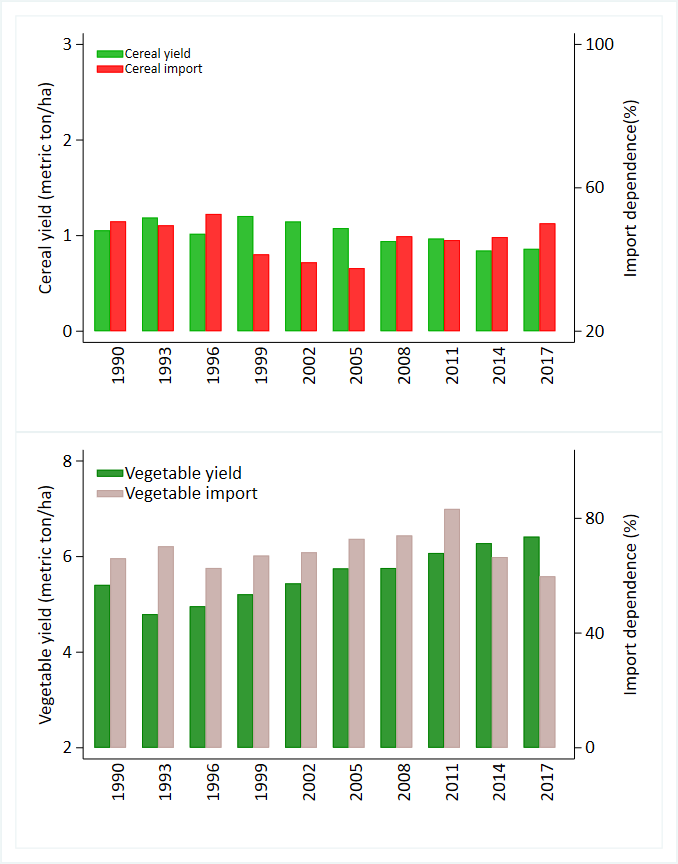


**Figure S4:** Relationship between crop yield and import dependency in The Gambia (1990-2017)

**Table S4:** Pairwise correlations of interrelationships between food system components in The Gambia

|  | Vegetable yields | Cereal yields | Employment in agriculture | GDP growth | Remittance | Urbanization | Iron deficiency | Vitamin A deficiency | Obesity |
| --- | --- | --- | --- | --- | --- | --- | --- | --- | --- |
| Vegetable yields | 1 |  |  |  |  |  |  |  |  |
| Cereal yields | **NS** | 1 |  |  |  |  |  |  |  |
| Employment in agriculture | **-0.983(<0.001)** | **0.646(0.004)** | 1 |  |  |  |  |  |  |
| GDP growth | 0.025(0.999) | -0.191(0.999) | -0.195(0.999) | 1 |  |  |  |  |  |
| Remittances | NS | NS | **-0.867((<0.001)** | 0.007(0.999) | 1 |  |  |  |  |
| Urbanization | NS | NS | **-0.984(<0.001)** | 0.286(0.999) | **0.858(<0.001)** | 1 |  |  |  |
| Iron deficiency | -0.054 (0.999) | 0.176 (0.999) | 0.048 (0.999) | 0.071 (0.999) | -0.365 (0.999) | 0.241 (0.999) | 1 |  |  |
| Vitamin A deficiency | **-0.852 (<0.001)** | **0.580 (0.034)** | **0.978 (<0.001)** | -0.276 (0.999) | **-0.832 (0.003)** | -0.998**(<0.001)** | NS | 1 |  |
| Obesity | NS | NS | **-0.996 (<0.001)** | 0.298 (0.999) | **0.851 (0.003)** | **0.982(<0.001)** | NS | NS | 1 |

Co-efficient (p-value)

P-values are Bonferroni corrected for multiple testing.

Bolded values indicate coefficients with p<0.05.

NS: Not supported by theorized pathway analysis.

**
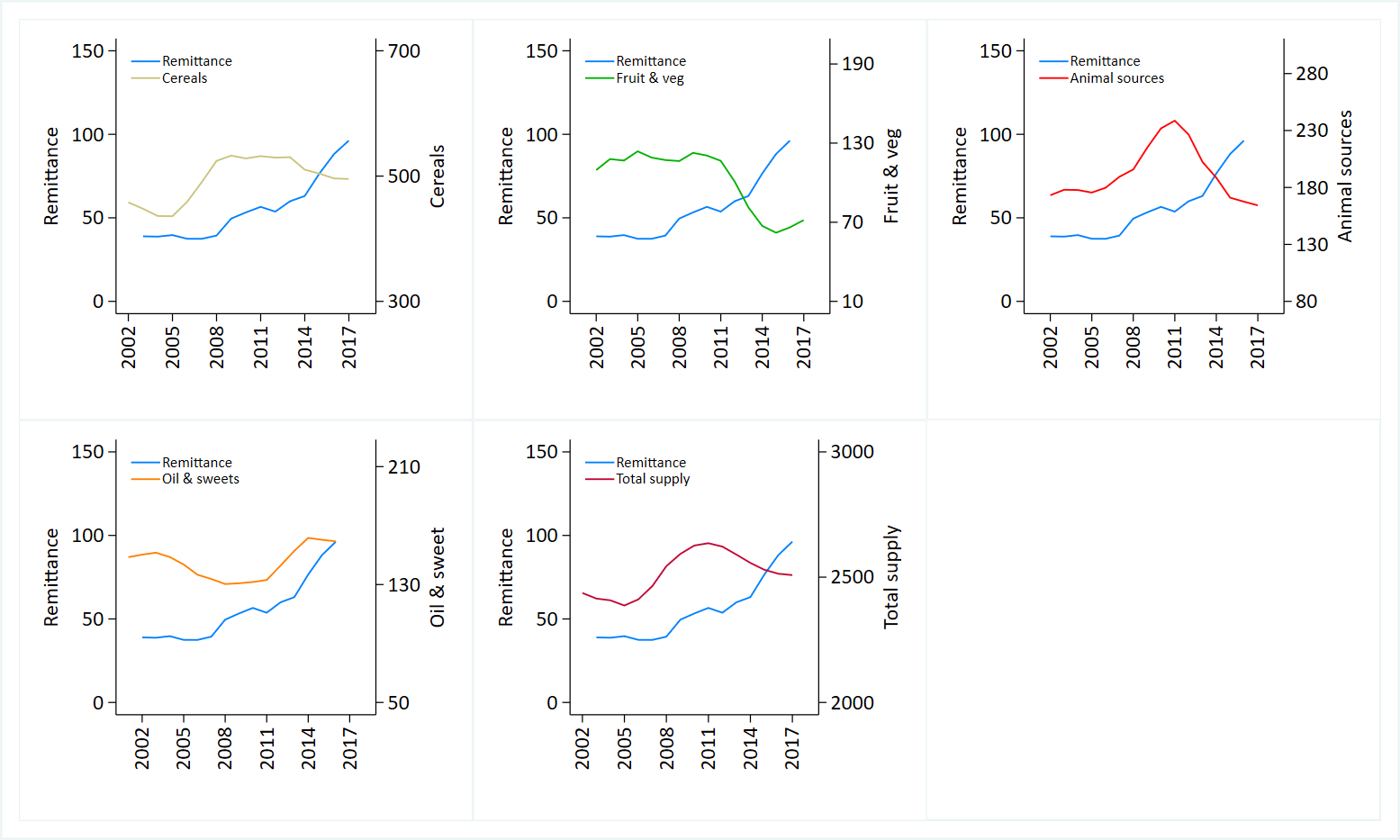
**

**Figure S5**: Remittances and food supply in The Gambia (1990-2017) [The specific food groups are in g/person/day; total supply is in kcal/person/day; remittance is in USD/person/year; GDP is in USD/year]


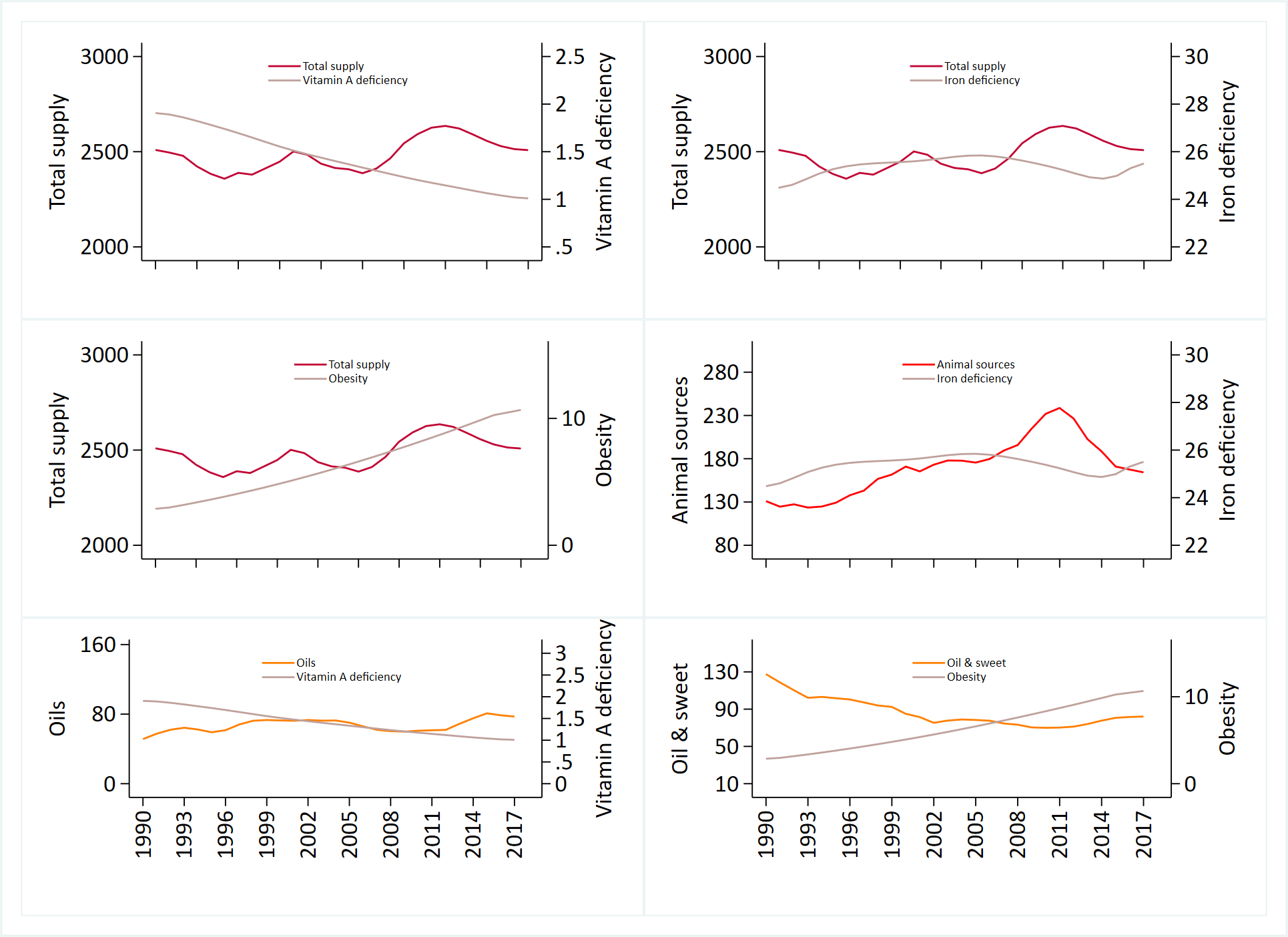


**Figure S6**: Supply of selected food groups (and energy) with nutrition and health outcomes in The Gambia (1990-2017) [Iron deficiency, vitamin A deficiency and obesity values are age-standardised prevalence estimates; the specific food groups are in g/person/day; total supply is in kcal/person/day].
